# Supplementary material for: Can bone scintigraphy reflect the progression of osteoarthritis after unicompartmental knee arthroplasty?
Source: PLoS One. 2023 Nov 8;18(11):e0288616. doi: 10.1371/journal.pone.0288616 (PMC10631669; doi:10.1371/journal.pone.0288616)
Supplement: Attachment — Submitted filename: Response to reviewers.docx [file pone.0288616.s002.docx]

| **Reviewer 1 Comments** | |
| --- | --- |
| Reviewer # 1’s Comments | The statement made in line 50-53 requires citation. |
| Author’s Answer | Thank you for taking your time and meticulously reviewing our manuscript. We have found your comments very professional and believe that improved our manuscript in many ways. We have done our best to answer your comments.  We have added additional citations as you recommended. |
| Changes made in manuscript | **Please refer to the ‘Introduction’ section (Pages 3, lines 46-49):** The imaging modality options for detecting such complications are narrow as lateral radiographs may show obscuration caused by the implant. Furthermore, magnetic resonance imaging (MRI) and computed tomography arthrography are limited by prosthetic interference [9-11]. |
| Reviewer # 1’s Comments | The statement made in line 64 requires citation. |
| Author’s Answer | We have added additional citations as you recommended. |
| Changes made in manuscript | **Please refer to the ‘Introduction’ section (Pages 4, lines 60-61):**  Furthermore, an increased uptake on BS may reflect degenerative changes in the knee [15, 21, 22] ... |
| Reviewer #1’s Comments | Clear study background and good flow of writing  Clear objectives and study hypothesis  Clear study selection criteria |
| Author’s Answer | Thank you for your comments. |
| Reviewer #1’s Comments | The statement made in line 96-101 requires citation, i.e., [18] |
| Author’s Answer | We have added citation as your recommendation. |
| Changes made in manuscript | **Please refer to the ‘Materials and methods’ section (Pages 5, lines 92-97):** K-L grade one was defined as doubtful narrowing of joint space and possible osteophytic lipping; grade two, as definite osteophytes and possible narrowing of joint space; grade three, as moderate multiple osteophytes, definite narrowing of joint space, and some sclerosis and possible deformity of bone ends; and grade four, as large osteophytes, marked narrowing of joint space, severe sclerosis, and definite deformity of bone ends [23]. |
| Reviewer #1’s Comments | Line 104-109 can be incorporated into a single sentence with citation or provide citation at the end of following sentence too, i.e. [21-22] |
| Author’s Answer | We have rewritten it in one sentence as recommended. |
| Changes made in manuscript | **Please refer to the ‘Materials and methods’ section (Pages 5, lines 100-105):** OA progression was defined as a K-L grade of one or higher and an OARSI atlas score of two or higher and was classified as "definitely progressed" (when the K-L grade was greater than one and the OARSI atlas score was greater than two), "possibly progressed" (when the K-L grade was equal to one and the OARSI atlas score was equal to two), or “not progressed” (when the K-L grade was lesser than one and the OARSI atlas score was lesser than two) [26, 27]. |
| Reviewer #1’s Comments | Both sentences, from line 111-117, requires citation |
| Author’s Answer | We have added citation as your recommendation. |
| Changes made in manuscript | **Please refer to the ‘Materials and methods’ section (Pages 6, lines 108-114):** Bone scan images of both knees (anteroposterior, posteroanterior, and lateral views) were obtained on a gamma camera (ORBITER, Siemens, Germany) 4 h after injection of 99mTechnetium-labelled 2,3-di carboxy propane-1, 1-diphosphonate (99mTc-DPD) (20mCi). Positive BS uptake was defined as uptake on either the femoral or tibial side of the contralateral compartment or the patellar or trochlear side of the patellofemoral compartment. BS uptake was graded according to the intensity of bone scintigraphic radiolabel retention (zero to two: zero = normal; one = mild; and two = intense) [28]. |
| Reviewer #1’s Comments | Flow of participants using a flow-chart would be better |
| Author’s Answer | Thank you for your comment. We have added a flow chart with Figure 1. |
| Changes made in manuscript | **Please refer to the ‘Figures’ section (Pages 24) :** Fig 1. Flow diagram shows the included patients that met the study criteria. |
| Reviewer #1’s Comments | Information regarding the occurrence of aseptic implant loosening would be interesting |
| Author’s Answer | Thank you for your valuable comment. Given the known association between aseptic implant loosening and BS uptake, we believe it would be possible to analyze this relationship in our patients as well. However, the aim of this study was to analyze the relationship between BS uptake in the remaining contralateral compartment and patellofemoral compartment after UKA and OA progression. Unfortunately, we were unable to conduct evaluations of BS in the surgically treated compartment and aseptic loosening. We will consider planning additional research to address this aspect in the future. |
| Reviewer #1’s Comments | Avoid repetition (similarly written in introduction) |
| Author’s Answer | We have taken your suggestions into account and revised the discussion section, making sure to avoid repetition and invest more time in its improvement. |
| Changes made in manuscript | **Please refer to the ‘Discussion’ section** |
| Reviewer #1’s Comments | line 162-68 can be deleted to establish a flow |
| Author’s Answer | As you suggested, this paragraph has been deleted. |
| Reviewer #1’s Comments | line 183-85: present study observed significant correlation between retained compartment OA progression compared to that reported by Misir et al. As this is the significant finding, further reasoning of this particular outcome is required. It can either be achieved by critical evaluation of Misir et al. study or identifying the reasons behind such significant correlation in the present study |
| Author’s Answer | Thank you for your comment. We further discussed the reasons for our results; however, we were unable to conclusively prove these differences. It is anticipated that further research will be needed in the future to investigate these aspects. Nevertheless, we believe that this study can serve as a reference for future investigations. |
| Changes made in manuscript | **Please refer to the ‘Discussion’ section (Pages 8, lines 172-182):** Additionally, Misir et al. [26] reported no correlation between patellofemoral and contralateral OA progression after UKA. However, our study found a significant correlation between the two as measured by radiography in both grading systems, but no correlation with BS uptake was observed. It is known that several factors are associated with the progression of OA after UKA [2, 26, 37, 38]. But there have been few reports regarding relationship OA progression between contralateral and patellofemoral compartment [26]. This study only included patients who had BS after UKA surgery, and the small number of patients may have contributed to the differing results compared to the previous study by Misir et al. Nevertheless, in the future, it is believed that this study can serve as a reference for understanding the relationship of OA progression in the remaining two compartments after UKA. |
| Reviewer #1’s Comments | Discussion section needs improvement. Author should give more effort in establishing the strengths and implications of study findings. It is also important to compare or contrast with the findings of previous study based on their methodological quality. |
| Author’s Answer | Thank you for your valuable comment. We have taken your suggestions into account and revised the discussion section, making sure to avoid repetition and invest more time in its improvement. |
| Changes made in manuscript | **Please refer to the ‘Discussion’ section** |

| **Reviewer 2 Comments** | |
| --- | --- |
| Reviewer # 2’s Comments | It is good to know with this study that OA in other compartment and patellofemoral OA progression occurs after few years of UKA. On top of that from this study it is known that patellofemoral OA does not alter clinical outcome. So UKA indications expanded to patient with Patellofemoral OA too. But it is better to search for better non invasive procedures prospectively to see OA progression with larger samples. Anyway I congratulate authors who have given their great efforts in preparing this article with good methods and methodology, adequate sample sizes, valid statistical tools and scientific analysis. I recommend prospective study with larger sample sizes performed by single surgeon in definite age group which might remove biasness regarding surgeon related mechanical failure cases. |
| Author’s Answer | Thank you for taking your time and meticulously reviewing our manuscript. We agree with your comments in that further prospective studies are required to further elucidate the role of bone scintigraphy after UKA. We are planning a randomized prospective study regarding bone scintigraphy after UKA in the future, as you have commented. Furthermore, we have made additional revisions focusing on the discussion section. |
